# Automatic Fracture Detection Convolutional Neural Network with Multiple Attention Blocks Using Multi-Region X-Ray Data

**DOI:** 10.3390/life15071135

**Published:** 2025-07-18

**Authors:** Rashadul Islam Sumon, Mejbah Ahammad, Md Ariful Islam Mozumder, Md Hasibuzzaman, Salam Akter, Hee-Cheol Kim, Mohammad Hassan Ali Al-Onaizan, Mohammed Saleh Ali Muthanna, Dina S. M. Hassan

**Affiliations:** 1 Institute of Digital Anti-Aging Healthcare, Inje University, Gimhae-si 50834, Republic of Korea; sumon39.cst@gmail.com (R.I.S.); arifulislamro@gmail.com (M.A.I.M.); salma05.eu@gmail.com (S.A.); 2 Software Intelligence, Dhaka 1229, Bangladesh; ahamadmejbah@gmail.com; 3 National Cancer Center, 323 Ilsan-ro, Goyang-si 10408, Republic of Korea; mdhasibuzzaman321@gmail.com; 4 Department of Intelligent Systems Engineering, Faculty of Engineering and Design, Middle East University, Amman 11831, Jordan; m.alonaizan@meu.edu.jo; 5 Department of International Business Management, Tashkent State University of Economics, Tashkent 100066, Uzbekistan; muthanna@sfedu.ru; 6 Department of Information Technology, College of Computer and Information Sciences, Princess Nourah bint Abdulrahman University, P.O. Box 84428, Riyadh 11671, Saudi Arabia; dshassan@pnu.edu.sa

**Keywords:** fracture, attention module, CNN, CBMA, multi region

## Abstract

Accurate detection of fractures in X-ray images is important to initiate appropriate medical treatment in time—in this study, an advanced combined attention CNN model with multiple attention mechanisms was developed to improve fracture detection by deeply representing features. Specifically, our model incorporates squeeze blocks and convolutional block attention module (CBAM) blocks to improve the model’s ability to focus on relevant features in X-ray images. Using computed tomography X-ray images, this study assesses the diagnostic efficacy of the artificial intelligence (AI) model before and after optimization and compares its performance in detecting fractures or not. The training and evaluation dataset consists of fractured and non-fractured X-rays from various anatomical locations, including the hips, knees, lumbar region, lower limb, and upper limb. This gives an extremely high training accuracy of 99.98 and a validation accuracy 96.72. The attention-based CNN thus showcases its role in medical image analysis. This aspect further complements a point we highlighted through our research to establish the role of attention in CNN architecture-based models to achieve the desired score for fracture in a medical image, allowing the model to generalize. This study represents the first steps to improve fracture detection automatically. It also brings solid support to doctors addressing the continued time to examination, which also increases accuracy in diagnosing fractures, improving patients’ outcomes significantly.

## 1. Introduction

Worldwide, millions of people of all ages and demographics suffer from bone fractures each year [1]. Fractures constitute a substantial burden on healthcare systems worldwide, ranging from falls among the elderly to sports injuries in young individuals [2]. A fracture diagnosis must be made quickly and accurately to start treatment on time, avoid complications, and promote the best possible recovery [3]. Radiographic imaging, such as X-rays, is used in clinical practice to detect bone fractures. This architecture offers pathologists and automated systems a viable method for diagnosing and grading breast cancer’s aggressiveness [4]. Deep learning transforms medical image processing by providing sophisticated pathological image analysis capabilities. It can recognize subtle features and patterns in images using advanced neural networks, enabling the early and accurate detection of various illnesses. This technology outperforms conventional methods in its field, with faster and more reliable results. Deep learning is, therefore, becoming crucial to modern pathology, improving patient care and diagnostic accuracy [5,6]. Deciphering X-ray images, however, may be challenging and time-consuming; qualified radiologists must carefully review each image to make a diagnosis. The need for automated systems to identify bone fractures is becoming more obvious.

**Figure 1:**
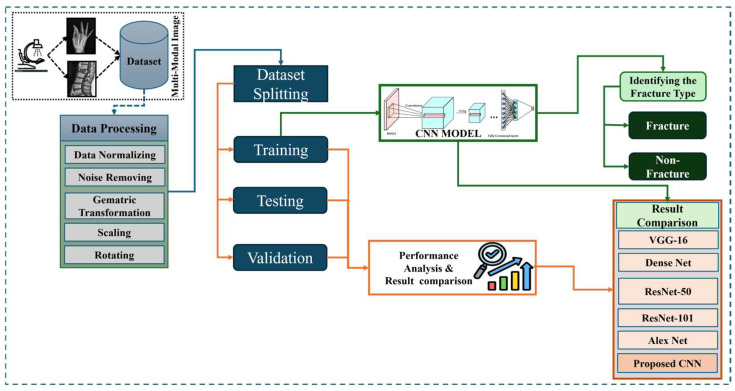
Flow diagram of automatic fracture detection.

Figure 1 shows the wide workflow of the proposed automatic fracture detection system using integrated CNN-based deep learning approaches with several attention mechanisms. The process begins with a diverse dataset of X-ray images collected from various physiological areas, including the hips, knees, lumbar spine, and limbs. These images undergo essential preprocessing stages such as generalization, noise removal, geometric changes, scaling, and rotation to increase quality and stability. After preprocessing, the dataset is divided into training, verification, and test sets. The system’s core is a convolutional neural network (CNN) model enhanced with a squeeze-and-excitation block and a convolutional block attention module (CBAM), allowing the model to focus on the main fracture features. The trained model classifies images into fractured or non-fractured categories. Finally, the system’s performance is evaluated and compared against current architectures such as VGG-16, Dense-Net, ResNet-50, ResNet-101, and Alex-Net, showing the superiority of the proposed CNN in accuracy and clinical efficiency.

Automatic models can potentially improve diagnostic accuracy, shorten interpretation times, and relieve the workload of medical practitioners by utilizing advances in AI and machine learning [7]. These devices can quickly and reliably analyze X-ray images, identify possible fractures for radiologists to examine further, or offer prompt preliminary evaluations in urgent care settings [8]. As reported in this article, constructing a strong automatic model for bone fracture identification aims to enhance patient outcomes by optimizing the diagnostic process. This study addresses the diversity and complexity of fractures encountered in clinical practice using a dataset comprising X-ray images of fractured and non-fractured anatomical regions, including the lower and upper limbs, lumbar region, hips, and knees [9]. The training, testing, and validation sets are carefully separated, offering a strong basis for the proposed automatic fracture identification system. Applying this automated detection approach could completely transform clinical operations and patient care. Improved diagnostic efficiency allows practitioners to improve treatment outcomes and intervene more quickly [10]. Deep learning has transformed medical image analysis, especially convolutional neural networks (CNNs), which make it possible to automatically, accurately, and quickly interpret complicated visual data. CNNs are very good at identifying minor elements in medical imaging, including anomalies or disease signs, since they collect spatial hierarchies within images [11,12]. CNNs are essential in many diagnostic applications, such as organ segmentation, fracture recognition, and tumor detection, due to their capacity to learn from large datasets [13,14]. As a result, CNN-based models are helping medical practitioners identify patients more quickly and accurately [15].

Additionally, this technology can be a helpful decision-support tool in emergencies or environments with limited resources, guaranteeing that patients receive timely and accurate assessments even in trying situations. Developing and validating an autonomous bone fracture detection model represent a significant advancement in medical imaging technology. Through artificial intelligence, this work contributes to ongoing efforts to enhance patient management and healthcare delivery in orthopedics and beyond [16]. The rest of this paper is organized as follows: Section 3 details the methodology of the deep learning approach. Section 4 presents the experimental setup and evaluation results, followed by a conclusion and discussion in Section 5 and Section 6.

## 2. Literature Review

The application of deep learning, specifically convolutional neural networks (CNNs), in medical image research has grown enormously in recent years, with fracture detection as a major research area. Several investigations have demonstrated the prospects of CNNs in automating fracture diagnosis, facilitating the avoidance of diagnostic errors, and improving efficiency in clinical workflows. To classify fractures from X-ray images, early fracture detection algorithms employed handmade features and conventional machine learning approaches like Support Vector Machines (SVMs) and Random Forests [17]. Regardless, variability in fracture forms, anatomical arrangements, and imaging requirements repeatedly introduced challenges for these methods [18]. The advent of deep learning revolutionized medical imaging by enabling end-to-end learning of hierarchical features directly from raw pixel data. CNNs, in particular, have demonstrated tremendous success in fracture detection due to their capability to capture spatial reliance and subtle pathological patterns [19].

For example, Rajpurkar et al. [20] created a CNN-based model (CheXNet) to identify different lung diseases, suggesting that deep learning might perform as well as or better than radiologists. The viability of AI-assisted fracture diagnosis was also demonstrated by Olczak et al. [21], who demonstrated a deep learning system for wrist fracture identification that achieved good sensitivity and specificity. While CNNs have shown promise, issues including class imbalance, false positives, and fracture appearance variability call for more complex structures. By allowing the model to concentrate on important areas while blocking out unimportant background noise, attention mechanisms have become a potent tool for improving CNN performance [22]. Research by Yoon et al. [23] showed that CBAM-integrated CNNs perform better than conventional CNNs in tasks like tumor segmentation and fracture identification that call for fine-grained localization. Squeeze-and-excitation (SE) blocks have also been utilized to improve model sensitivity to key regions by recalibrating feature responses [24]. Most fracture detection models now in use are anatomically specialized and have only been trained on one location, such as the knee, hip, or wrist [25]. However, generalizable models that can identify fractures across several anatomical sites are necessary for real-world clinical circumstances, using better particle herd adaptation (IPSO) with a hybrid CNN and LSTM architecture, including cloud-based fault classification systems [26]. While these studies focus on high-voltage insulator diagnostics, they portray the growing ability of cloud-integrated and adaptable intensive learning structures to detect fractures in real time, with high compatibility [27].

Similarly, our work contributes to this domain by proposing a multi-delay CNN model to detect fractures in diverse physical X-ray data. Unlike pre-domain-specific applications, our model integrates SE modules and CBAM to increase spatial- and channel-wise feature attention and improve clinical precision [28]. Future work will incorporate cloud-preserving and metaheuristic optimization to further enhance scalability and utility for clinical purposes. Recent studies investigated multi-region fracture detection but encountered difficulties in maintaining high accuracy across various datasets [29]. We present a sophisticated CNN model for multi-region fracture detection with several attention blocks (CBAM and squeeze modules) to address these shortcomings. In this work, we trained our model on a diverse, multi-region X-ray dataset, which increases generality. We also propose a unique combination of SE and CBAM attention modules within a CNN framework, which is rarely seen in fracture detection. Additionally, the model achieves high accuracy with low complexity, making it suitable for real-world clinical applications. Past approaches often failed to normalize well for multi-sector data, and there was a lack of attention mechanisms to refine the convenience of the representation.

In contrast, our proposed model introduces a hybrid attention–comprehensive CNN architecture that integrates SE and CBAM, which enables better channel and spatial attention. This allows the model to highlight clinically relevant areas in diverse physical regions. Additionally, the model acquires high clinical accuracy with low computational complications, providing a practical and scalable solution for the real world. This experiment achieves 99.98% training and 97.72% validation accuracy, presenting the effectiveness of attention mechanisms in improving fracture detection. This study builds upon earlier research while introducing novel architectural enhancements to bridge the gap between AI and clinical applicability in fracture diagnosis.

## 3. Materials and Methods

### 3.1. Data Acquisition

The dataset used in this study consists of 10,580 radiographic (X-ray) images, including both fractured and non-fractured instances across various anatomical regions such as the lower limb, upper limb, lumbar region, hips, knees, and more. This comprehensive dataset is structured into three subsets: 9246 images for training, 828 for validation, and 506 for testing [30].

**Figure 2:**
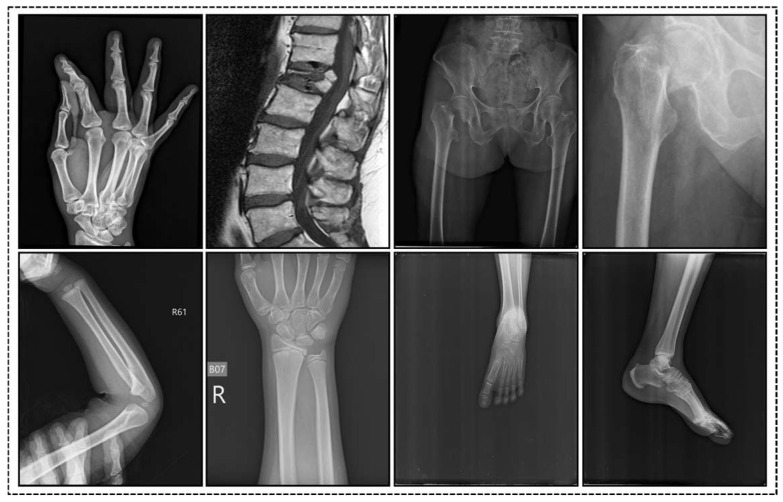
The first row displays sample images of fractures, while the second row shows sample images of non-fractures.

Such meticulous organization ensures a balanced and thorough evaluation of the proposed automatic fracture detection system. The dataset, which is publicly accessible on Kaggle, serves as a crucial resource for training and assessing the model’s performance in identifying fractures in clinical settings. Figure 2 shows fractured and non-fractured images of the training sample. 

### 3.2. Data Preprocessing 

A comprehensive preprocessing pipeline was implemented to ensure the integrity and stability of input data to train the proposed deep learning model. Initially, data generalization was applied by scaling the pixel intensity values to the range [0, 1], standardizing the image input, and facilitating rapid convergence during model training. Given the clinical nature of radiographic images, which are often susceptible to various forms of noise due to equipment variability or acquisition artifacts, noise techniques—especially Gaussian and Median Filtering—were employed to enhance image clarity without compromising significant structural details. A suite of geometric changes was introduced to improve the model’s generalizability and combat overfitting as part of the data growth strategy. These changes included horizontal and vertical flipping, random winding (within ±20 degrees), and scaling operations following real-world variations in fracture orientation and anatomical presentation. This enhanced the training dataset’s effective size and enabled the model to learn stronger and irreversible features. In addition, careful attention was given to maintaining physical purity during change, ensuring that the major clinical patterns were preserved. This careful preprocessing strategy played an important role in increasing the performance of the proposed fracture detection model, allowing it to effectively generalize to various patients and imaging situations faced in clinical practice.

### 3.3. Method

**Figure 3:**
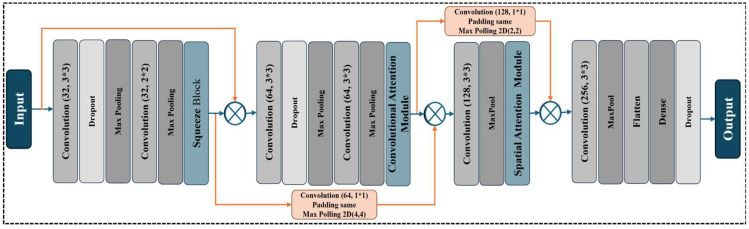
Backbone architecture of CNN with multiple attention modules. Orange color arrow indicate the residual connection.

The network architecture flow is illustrated in Figure 3. The figure sequentially maps the model components—from the input layer and initial conversion and dropout blocks to squeezed blocks, additional conversion layers, CBAMs, and spatial attention after modules. Skip connections and intermediate pooling are applied to increase the convenience of the flow and preserve spatial details. Finally, the architecture proceeds with high-dimensional determination and flattened, fully connected layers, causing output. It provides a transparent and systematic view of the design of step-by-step visual map models. Figure 3 presents the proposed backbone architecture for automatic fracture detection using X-ray images. This model is built on a convolutional neural network (CNN) enhanced with several attention mechanisms, with a skip to maintain the squeezing-and-excitation (SE) block, the convolutional block attention module (CBAM), and the spatial attention network to maintain desirable characteristics in the local attention network. The model begins with an input X $\in R^{H\times W\times C}$, where H, W, and C represent the image height, width, and number of channels, respectively (in this case, 128 × 128 × 3). The initial layers consist of convolutional feature extractors to obtain low- and mid-level representations, formulated as $F^{1}$ = *Relu (Con2D(X,*$F_{1}$*)),* followed by regularization and spatial reduction using $F^{2}$ = *MaxPool2D (Dropout(*$F^{1}$*)).* These convolutional blocks, comprising two layers with 32 filters each, are succeeded by a squeeze-and-excitation (SE) block that adaptively recalibrates channel-wise feature responses. For an input feature map *U*$\in R^{H\times W\times C}$, the SE block performs a squeeze operation via global average pooling:

(1) $$z_{c}=\frac{1}{H\times W}\sum_{i=1}^{H}.\sum_{j=1}^{W}U_{c}(i,j)$$

and an excitation operation through fully connected layers and nonlinearities: s = σ *(*$W_{2}.{\delta (W}_{1}.z$*))*, where δ denotes ReLU, σ denotes sigmoid activation, and s is the resulting scale vector. The input is then reweighed as ${û}_{c}$ = $s_{c}.U_{c}$. Subsequent convolutional layers use 64 filters, and their output is fed to the convolutional block attention module (CBAM), which gradually applies channel and spatial attention. 

The attention of the channel is calculated by combining the average maximum pooling operations with the local dimension; then, the results are passed through a shared multi-layer perceptron (MLP):

(2) $$U_{c}=\sigma \left(MLP\left(AvgPool\left(F\right)\right)+MLP\left(MaxPool\left(F\right)\right)\right)$$

where $M_{c}\in R^{1\times 1\times C}$. This is followed by spatial attention, which refines the spatial locations via

(3) $$M_{s}=\sigma (f^{3\times 3}([AvgPool\left(F\right);MaxPool(F)]))$$

where $M_{s}$ $\in R^{H\times W\times 1}$ and $f^{3\times 3}$ denote a convolution with a 3 × 3 kernel. The attention-refined output is calculated as

(4) $$F^{\prime }=M_{c}\left(F\right).F,\;followed\;by\;F^{\prime \prime }=M_{c}\left(F^{\prime }\right).F^{\prime }.$$

Residual skip connections are introduced across attention and convolutional blocks to ensure deeper feature reuse and mitigate vanishing gradients. These connections are resized to match the target dimensions using 1 × 1 convolutions and max pooling:

(5) $${Skipl}_{adj}=MaxPool\left(Conv{2D}_{1\times 1}\left(SE_{Output}\right)\right)$$

and are incorporated as $x=ReLU(x+{Skipl}_{adj})$. After a convolutional block with 128 filters, spatial attention is further applied to refine informative spatial locations, followed by an additional skip connection from the CBAM block to the output of the spatial attention block.

The final stages of the architecture include a firm layer with 256 filters, global flattening, and a densely associated layer with dropouts. The final layer is a single neuron with sigmoid activation. The final binary classification output is $y^{\prime }=\sigma (W_{Out}.x+b)$, where $y^{\prime }\in [0,1]$ reflects the estimated possibility of fracture. The model is ready to highlight and preserve important characteristics to detect strong fractures in attention-directed and residual-derived architectures, especially in medical radiography.

### 3.4. Squeeze Block 

**Figure 4:**
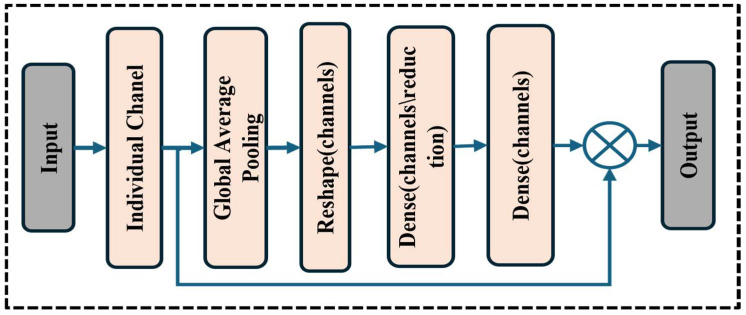
Squeeze block.

In our methodology, as illustrated in Figure 4, we incorporated a squeeze block to enhance the feature representation capabilities of our deep learning model for automated bone fracture diagnosis using X-ray images. Using a sequence of dense layers and global average pooling, the squeeze block is intended to adjust channel-wise feature responses selectively [31]. First, to determine the relative relevance of each feature channel, feature maps are aggregated across spatial dimensions using the global average pooling procedure.

The squeeze block computes channel-wise attention scores after reshaping and dimensionality reduction using dense layers with ReLU activation. A sigmoid activation layer produces these scores, which indicate each channel’s significance for feature recalibration. In summary, by multiplying the original feature tensor by the estimated attention scores, the SE block amplifies relevant information and suppresses less relevant information. This adaptive recalibration procedure greatly improves our model’s discriminative capability, facilitating the detection of fractures in various clinical scenarios and anatomical locations.

### 3.5. Convolutional Block Attention Module (CBAM)

**Figure 5:**
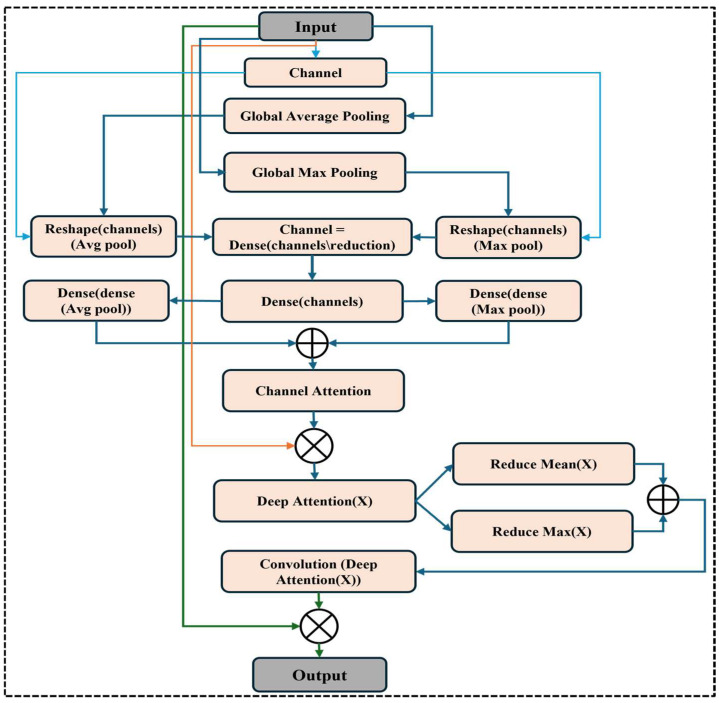
Convolutional block attention module of backbone architecture.

The convolutional block attention module (CBAM) in Figure 5 enhances the feature representation in the bone fracture multi-region X-ray data analysis context. Using global average pooling (GAP) and global max pooling (GMP), the CBAM block initially applies channel attention to concentrate on the most informative channels. The GAP and GMP results are specifically modified and routed through dense layers with a reduction factor, usually set to 16, to compactly represent the channel features before restoring the original channel count. CBAM is an attention method that improves feature representation by sequentially applying channel and spatial attention. Channel attention, which focuses on finding the most useful channels, is used first in CBAM. Next, spatial attention is applied, highlighting important spatial areas in the feature maps. Thanks to this two-step procedure, the network can attend to both “what” and “where” in terms of its focus on a picture, which is particularly helpful for complicated visual patterns like those found in medical scans.

For each feature channel, the channel attention method first creates two unique descriptors using global max pooling (GMP) and global average pooling (GAP), respectively. Considering a feature map input, *F*$\in R^{H\times W\times C}$, where H,W, and C are the image height, width, and number of channels, respectively,

(6) $$f_{c}^{avg}=GAP(F)+\frac{1}{H\times W}=\sum_{i=1}^{H}\sum_{j=1}^{W}F\left(i,j,c\right)$$

(7) $$f_{c}^{max}=GAP\left(F\right)=\underset{i,j}{\max}F(i,j,c)$$

where the average- and maximum-pooled features for the channel C are denoted by $f_{c}^{avg}$ and $f_{c}^{max}$. Channel attention weights are then calculated by processing these descriptors through a shared multi-layer perceptron (MLP). To restore the original channel dimensions, the MLP comprises an expansion layer after a reduction layer (with a reduction ratio of r). The result is calculated as follows:

(8) $$M_{C}(F)=\sigma \left(MLP(\int_{c}^{avg})+\;MLP(\int_{c}^{max})+\;)\right)$$

Here, σ is the sigmoid activation function, and $M_{C}$(F)$\in R^{1\times 1\times C}$ is the channel attention map. The channel attention map is then used to scale the original feature map. F: $F^{\prime }$ = $M_{c}$*(F). F.* The channel-refined feature map’s key spatial areas are then highlighted using deep attention. Deep attention is accomplished by calculating two spatial descriptors across the channel dimension, average pooling, and max pooling.

(9) $${\;\;\;f}_{spatial}^{avg}=\frac{1}{C}=\sum_{C=1}^{C}F^{\prime \left(i,j,c\right)}$$

(10) $$f_{spatial}^{max}=\underset{C}{\max}F^{\prime \left(i,j,c\right)}$$

where $f_{spatial}^{avg}$ stands for the average spatial feature and $f_{spatial}^{max}$ for the maximum-pooled spatial feature. The deep attention map is created by concatenating these deep descriptors along the channel dimension and passing them through a convolutional layer with a 7 × 7 kernel size.

(11) $$M_{d}\left(F^{\prime }\right)=\sigma \left({Conv}_{7\times 7}\left(\left[f_{spatial}^{avg},f_{spatial}^{max}\right]\right)\right)$$

where the sigmoid function is indicated by σ, and the deep attention map is represented by $M_{d}$(F)$\in R^{1\times 1\times C}$. The channel attention module’s feature map is then scaled using the spatial attention map: $F^{\prime \prime }=M_{s}$*(F*′*). F.* CBAM’s output, F, improves the network’s focus on pertinent channel relevance and spatial importance features. This module enhances the network’s capacity to identify fractures in X-ray pictures by combining channel and spatial attention.

### 3.6. Spatial Attention Modules 

Within the bone fracture multi-region X-ray dataset, the SAB improves the localization of important features. It uses average and maximum pooling operations across the channel axis to identify and highlight significant spatial regions in the input tensor [32,33].

**Figure 6:**
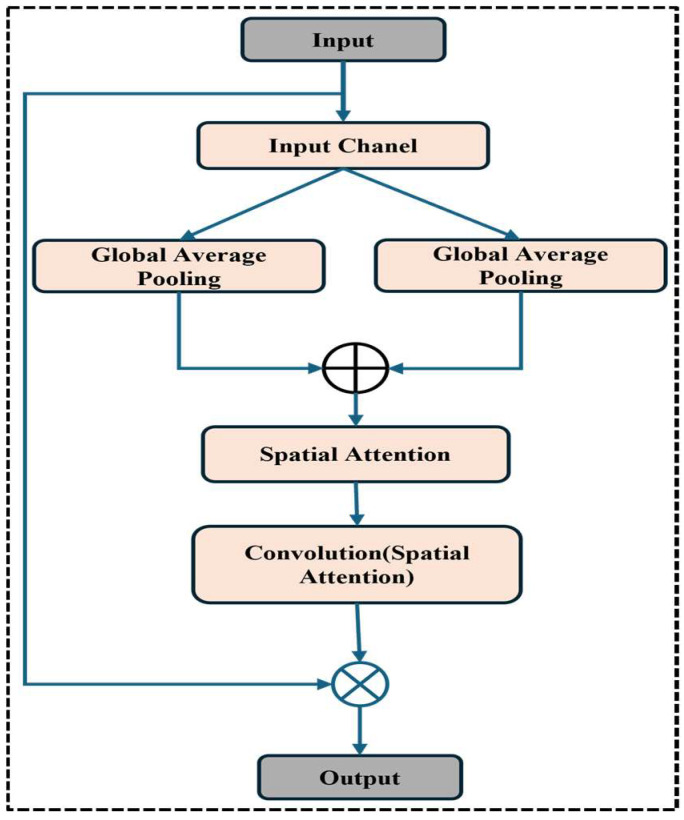
Spatial attention block.

The block first computes the max-pooled and average-pooled feature maps, obtaining crucial spatial information, shown in Figure 6. These pooled feature maps are subsequently concatenated along the channel axis to create a thorough depiction of the spatial context. After passing through a convolutional layer with a 7 × 7 kernel, this concatenated feature map is processed via a sigmoid activation function to produce a spatial attention map. The input tensor is scaled using this spatial attention map by element-wise multiplication, emphasizing the most pertinent spatial regions suggestive of fractures. The model can better focus on the areas within the X-ray images where fractures are likely to occur. This approach enhances the fracture detection system’s overall diagnostic accuracy and robustness.

## 4. Experiment Results

**Figure 7:**
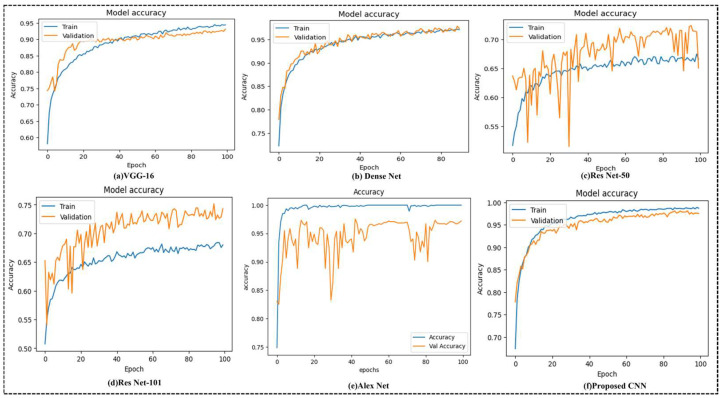
The training accuracy and validation accuracy of the six different algorithms, which are represented graphically: (**a**) VGG-16, (**b**) Dense Net, (**c**) Res Net-50, (**d**) Res Net-101, (**e**) Alex Net, and (**f**) proposed CNN.

This study’s automated bone fracture detection model achieved exceptional performance across all evaluation metrics, demonstrating its efficacy and reliability in clinical applications. Training the model on a comprehensive dataset of fractured and non-fractured X-ray images from diverse anatomical regions yielded remarkable results. The model successfully learned to differentiate between images that depict fractures of the lower and upper limbs, the lumbar region, the hips, the knees, and other body parts, and those that do not. The model’s capacity to reduce errors while learning is further demonstrated by its minimal training loss (0.0010). The model’s generalizability and robustness were validated. The validation accuracy, 96.72%, demonstrated the model’s capacity to identify fractures in previously unknown X-ray images, as shown in Figure 7. To assess the effectiveness of our model during the experimentation phase of this work, we used confusion matrices together with several related metric measures, including accuracy (Acc), precision (Pre), recall (Rec), the F1-score (F-Score), and the Cohen Kappa score (Ckp). The confusion matrix’s true positive (TP), false positive (FP), true negative (TN), and false negative (FN) parameters were used to calculate these measurements. The confusion metrics were calculated using the following formulas:

(12) $$Acc=\frac{TP+TN}{TP+TN+FP+FN}$$

(13) $$Pre=\frac{TP}{TP+FP}$$

(14) $$Rec=\frac{TP}{TP+FN}$$

(15) $$F1-Scr=\frac{2*(Rec*Pre)}{\left(Rec+Pre\right)}$$

(16) $$Ckp=\frac{P_{0}-P_{e}}{1-P_{e}}$$

$P_{0}$ indicates that there is a high percentage of observed agreement between raters and classifiers. $P_{e}$ is the hypothetical probability of chance.

To determine the effectiveness of our proposed model, we compared its implementation against several widely used deep learning architectures, including VGG-16, DenseNet, ResNet-50, ResNet-101, and AlexNet. Table 1 demonstrates the evaluation metrics of all models on the test set, which includes classification accuracy, precision, recall, the F1-score, Cohen’s Kappa (Ckp) score, the number of trainable parameters, model complexity, and total training time. The proposed attention-based CNN model exceeded all baseline architectures regarding classification performance. It gained the highest training accuracy of 99.98% and a test accuracy of 96.72%, indicating strong detection capabilities across multiple anatomical X-ray regions. In terms of precision and recall, the model showed a remarkable balance with 98.12% precision and 95.00% recall, leading to an F1-score of 97.00%. The Cohen’s Kappa score of 96.39% indicates excellent agreement beyond chance between the predicted and actual labels. While AlexNet achieved a slightly lower test accuracy (95.65%) and F1-score (94.97%), it required a considerably longer training time of 35,000 s and had a larger parameter count (4.67 million). Dense Net, known for its efficient parameter usage, showed competitive performance with 94.38% test accuracy and a 95.17% F1-score while falling short of the proposed model in most evaluation criteria. Our proposed model has a parameter count (1.58 million) and a reasonable training time (16,000 s), significantly outperforming others in diagnostic performance. Including CBAM and squeeze attention blocks likely contributed to its improved focus on relevant fracture features across diverse anatomical regions. These results demonstrate the proposed model’s robustness, efficiency, and excellent diagnostic ability, emphasizing its potential as an assistive tool in clinical environments for automatic fracture detection across multi-region X-ray data compared to traditional techniques like VGG-16, RESNET-50, RESNET-50, RESNET-50, DENSENET, and AlexNet, which are in the major matrix (accuracy, precision, recall, F1-score, and Cohen Kappa). These additions reveal the performance benefits of our model. In addition, we have included a radar chart to visually compare all models, which displays the proposed CNN’s high and more consistent performance in several assessment criteria. These enhancers strengthen the clarity and impact of our performance analysis.

Figure 7 illustrates the training and validation accuracy curves for different deep learning models used for the multi-region bone fracture data: (a) VGG-16, (b) DenseNet, (c) ResNet-50, (d) ResNet-101, (e) AlexNet, and (f) the proposed CNN model with 100 epochs. The proposed CNN model (f) performs best and most consistently. It reaches a nearly excellent training accuracy of 99.98% and an increased validation accuracy of 96.72%, with smooth and steady confluence throughout the training epochs. The proposed model has learned the complex patterns within the X-ray images and generalized well to unseen validation data without overfitting. ResNet-50 and ResNet-101, on the other hand, display slower intersections and greater variability, implying less consistent learning behavior. Even if DenseNet performs poorly, these other models are still not as accurate and consistent as the suggested model. These findings demonstrate the proposed methodology’s clinical potential, accuracy, and robustness in automating fracture identification across various anatomical locations.

These curves reflect the performance of the model on a dataset of 10,580 X-ray images from diverse physical areas (hips, knees, lumbar, upper/lower limbs), with the highest validation of 96.72% achieved with the proposed CNN (f), which achieves an accuracy and stable conversion of 96.72%, indicating strong generalizations. In contrast, Resnet-50 (c) displays a verification accuracy of 65.02%, characterized by significant variability and slow convergence, indicating potential overfitting. This stability highlights the effectiveness of the integrated attention mechanisms, including the concentration module (CBAM) and squeezed blocks, in increasing the ability of the model to detect fractures in diverse physical areas correctly. The proposed CNN model incorporates several regularization strategies to reduce overfitting. First, the dropout layers with a rate of 0.3 are applied randomly, and after dense layers, they are used to reduce dependence on specific characteristics and increase generalizations during training.

**Figure 8:**
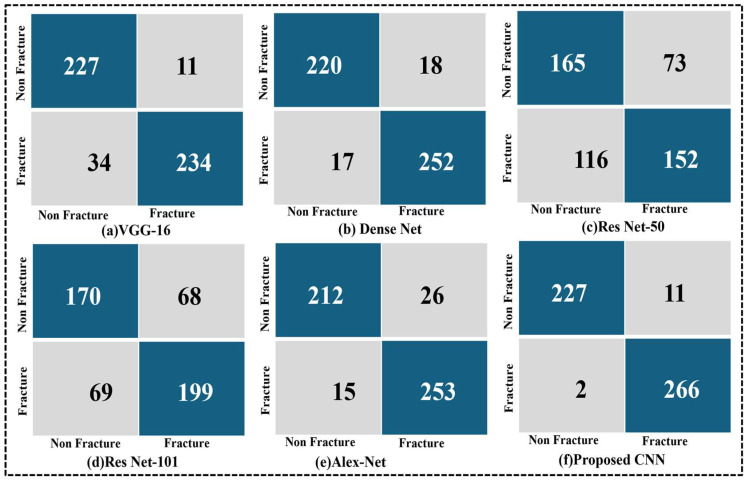
Comparison of confusion matrix (**a**) VGG-16, (**b**) Dense Net, (**c**) Res Net-50, (**d**) Res Net-101, (**e**) Alex Net, and (**f**) proposed CNN.

Comparisons of confusion matrices for VGG-16, Dense Net, ResNet-50, ResNet-101, Alex Net, and the suggested CNN in bone fracture classification are shown in Figure 8. The proposed CNN performs best with a balanced true positive-to-true negative ratio and few misclassifications. Conversely, ResNet-50 exhibits the highest misclassification rate, whereas VGG-16 and ResNet-101 have reasonable accuracy with a few false positives. These findings support the suggested CNN’s ability to detect fractures in various anatomical locations reliably. With just 11 false positives and two false negatives, as well as 227 true positives and 266 true negatives, the suggested CNN model (f) outperformed all other architectures regarding classification performance. This demonstrates the model’s resilience, low error rate, and great promise for precise and trustworthy diagnosis in medical image classification methods. It also shows excellent precision and recall.

We conducted coupled T-testing on the test set to compare major demonstration metrics including accuracy, recall, the F1-score, and Cohen’s Kappa score. Additionally, we applied the McNemar test to the confusion matrices (Figure 8), confirming that the proposed model’s low false rate is statistically important compared to other models (*p* < 0.05).

**Figure 9:**
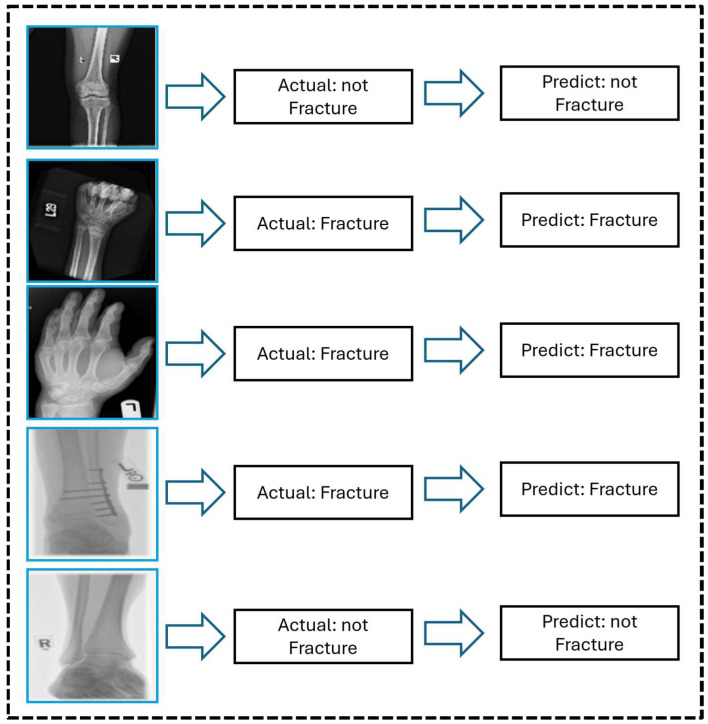
Proposed model visual prediction results.

Figure 9 shows the graphical prediction results generated by the proposed CNN model on X-ray images, emphasizing its ability to accurately classify fractured and non-fractured cases. Most predictions align accurately with the labels, revealing the model’s robustness for real-world techniques. The model’s importance in supporting clinical diagnosis is demonstrated by its notable ability to detect small fractures, which are sometimes difficult to recognize. Overall, this study’s results highlight the potential of automated bone fracture detection models to enhance diagnostic efficiency and accuracy in medical imaging. Figure 9 shows representative X-ray images with the model’s predicted classification results, both clear and fine, and the successful detection of clear and less-visible fractures. These examples highlight the model’s sensitivity for subtle dissection and structural variations in the areas of bones, especially in challenging cases where fractures are visually minimal and easily missed by the human eye. By incorporating these qualitative results, the proposed attention-based CNN results confirm the clinical reliability of the model and its potential utility in real-world clinical scenarios.

## 5. Discussion

This study proposes a novel CNN model integrating SE and CBAM attention modules for automated fracture detection. Unlike existing methods, which center on single areas, our model handles several anatomical areas within a structure. It is trained on a diverse X-ray dataset covering the hips, knees, spine, and organs. The model demonstrates high accuracy with low parameters to ensure performance and efficiency. This design addresses key gaps in generalization and clinical applicability. By leveraging advanced machine learning techniques, our model demonstrates significant progress toward supporting healthcare professionals in timely and accurate fracture diagnosis, ultimately improving patient outcomes and healthcare delivery. Continued research and development in this field promise further advancements, paving the way for more effective technological integration into clinical practice. The advantage of our model, integrating both SE blocks and CBAM, is that it enhances both channel-wise and spatial attention, allowing it to focus on the most relevant characteristics associated with fractures. This dual-focus strategy significantly improves the model’s ability to make micro-fracture patterns local, which can be remembered by a traditional CNN. The model was trained on a diverse, multi-field X-ray dataset (hips, knees, lumbar region, upper and lower limbs), which enables it to normalize in various physical structures. It contradicts many existing models limited to the same region and is less applicable to real-world clinical scenarios. The proposed network’s high classification performance (96.72% validation accuracy, 97.00% F1-score) enables a relatively low parameter count (1.58 million) and short training time with deep architectures such as ResNet-101 or AlexNet. This makes it computationally efficient and suitable for real-time or resource-limited clinical environments. This study systematically examined the performance of several deep learning architectures—VGG-16, DenseNet, ResNet-50, ResNet-101, AlexNet, and the proposed CNN model—on a medical image category assignment. The evaluation was conducted using training and validation accuracy curves (Figure 7) and confusion matrices (Figure 8), allowing a wide analysis of each model’s learning behavior and classification capabilities. The proposed CNN model demonstrated the best and most consistent performance during the training and validation stages. The proposed model demonstrated great learning efficiency and robust generalization without noticeable overfitting, as seen in Figure 7f, with a training accuracy of over 99% and a validation accuracy that was nearly aligned. Models such as ResNet-50 (Figure 7c) showed significant variance and discrepancies in validation accuracy, indicating fluctuation and possible overfitting problems. This emphasizes the proposed model’s reliability and precision in distinguishing between complex class allocations, making it a valuable solution for high-stakes applications such as medical diagnostics.

In summary, the proposed CNN architecture surpassed established state-of-the-art models in accuracy and robustness. Its superior learning curve and confusion matrix profile demonstrate its potential for deployment in real-world clinical environments, where precision and reliability are paramount. The outcomes demonstrate that a well-designed, task-specific CNN can outperform deeper and more complex pre-trained models when properly tuned and trained on domain-specific data.

The proposed CNN model was applied using an input resolution of 128 × 128 × 3, which balances computational efficiency and adequate spatial details for fracture localization. The network was trained using the Adam Optimizer with a learning rate of 0.001, which was chosen based on empirical verification in medical image classification works and its proven convergence stability. A batch size of 32 was used to maintain efficient GPU use while preserving generalization capacity. To prevent overfitting, dropout layers with a rate of 0.5 were applied after fully connected layers. The Relu activation function was employed in hidden layers, and sigmoid activation was used in the final output layer for binary classification. The number of filters in convolutional layers progressively increased (32, 64, 128, 256) to effectively remove both low- and high-level features. The significance of SE and CBAM was determined through ablation experiments, demonstrating their significant contribution to improving verification performance. All hyperparameters were adapted through recurrence and grid search, based on verification accuracy and training stability. These configurations reflect a carefully tuned design for multi-region X-ray fracture detection complications. A major limit is the dependence on the public dataset, which cannot accommodate a variety of clinical imaging conditions, such as different resolutions, noise levels, and patient demographics. This can affect the normality of models in various institutions or imaging devices.

Additionally, while the model performs well in binary classification (fracture versus non-fracture), it does not currently support fracture type localization or severity grading, which are important for clinical decisions. We plan to expand the model for future work by incorporating multi-class classification and fracture localization techniques using bounding boxes or segmentation maps. We aim to validate the model on an external, multi-institutional dataset to assess its strength in a broad clinical environment. In addition, integrating explainable AI techniques can help increase clinical interpretation and user trust.

## 6. Conclusions

In this study, we have explored the development and implementation of an automated bone fracture detection model using X-ray imaging to enhance diagnostic accuracy and streamline clinical workflows. Leveraging a dataset comprising fractured and non-fractured images across various anatomical regions, including the lower limb, upper limb, lumbar region, hips, and knees, our methodology involved rigorous training, testing, and validation phases. The Introduction highlighted the global prevalence of bone fractures and underscored the critical need for efficient and accurate diagnostic tools. Traditional methods of fracture detection rely heavily on radiographic interpretation by skilled professionals, which can be time-consuming and prone to variability. Our approach to developing an automated fracture detection model builds upon artificial intelligence and machine learning advancements. The results of our study suggest that our model can effectively detect fractures across various anatomical regions, offering consistent performance comparable to or exceeding that of human experts in preliminary assessments. These results provide a convincing path for using sophisticated computational methods in clinical settings, opening the door to more precise diagnoses and effective treatment plans. This research provides valuable insights into the process of developing computational techniques for the diagnosis of lung cancer. It also highlights the necessity for continued study and validation to develop clinical applications and the value of hybrid deep learning models in categorizing X-ray images. However, this study’s reliance on a single dataset for training and assessment may limit the model’s capacity to adapt to various clinical situations. Furthermore, deep learning architectures intended for medical image processing should be made simpler to optimize the computing economy without compromising diagnostic accuracy.

## Figures and Tables

**Table 1:** Evaluation metrics on the test set for classifying bone fracture multi-region X-ray data using different deep learning algorithms.

Machine Learning Model	Training Accuracy (%)	Testing Accuracy (%)	Precision (%)	Recall (%)	F1-Score (%)	Ckp Score (%)	Number of Parameters	Complexity	Training Time
VGG-16	94.39	93.12	87.00	95.00	91.01	90.99	4.50 m	O(n^2^)	28,000 s
Dense Net	95.98	94.38	95.38	96.10	95.17	95.38	0.77 m	O(n^2^)	11,000 s
ResNet-50	66.97	65.02	59.03	67.00	64.00	66.89	2.60 m	O(n^2^)	15,000 s
ResNet-101	68.99	74.12	71.12	71.09	71.12	71.25	4.60 m	O(n^2^)	29,000 s
Alex Net	99.10	95.65	94.99	95.89	94.97	95.11	4.67 m	O(n^2^)	35,000 s
Proposed CNN	99.98	96.72	98.12	95.00	97.00	96.39	1.58 m	O(n^2^)	16,000 s

## Data Availability

The dataset is publicly accessible on Kaggle.
